# Municipal Hospital and Clinical Benefits

**Published:** 1921-08-20

**Authors:** 


### Municipal Hospital and Clinical Benefits.

### A REGISTRATION FEE AND TREATMENT SCALE.

DR. GEORGE F. BUCHAN, the Medical Officer of Health for Willesden, has prepared a scale of charges for medical treatment, and in a general report on the subject he states, that in 1920, 995 expectant mothers, 2,731 nursing mothers, and 6,308 children under five, making a total of 10,034 maternity and child-welfare cases, made 51,468 attendances. School-children numbering 11,183 made 64,588 attendances. In addition, 4,894 persons made 12,493 dental attendances. Similarly 681 patients were nursed at home, on whom 11,531 attendances were made by the nurses.

If it is decided that a charge be made for attendance at the municipal clinics, the simplest plan would be to collect the charge by way of an annual registration fee. If this method were adopted, then the services to be rendered in respect of the charge should include the following:

The out-patient medical and dental care, supervision, and treatment of expectant mothers, nursing mothers, children under five, and school-children. The benefit should include the use of the clinics for expectant mothers, nursing mothers, babies, minor ailments, throat, nose, and ear, eye, ringworm, and dental; and also special consultations, prescriptions, and home nursing. The foregoing benefits would be received by all mothers and children under five resident in Willesden, and school-children attending the elementary schools, on payment of an annual fee of 2s. 6d.

As regards the municipal hospitals the benefits would include in-patient treatment in respect of confinements, diseases of women, and diseases of children up to school-leaving age. Charges for municipal-hospital benefits would be: Confinement cases, £2; operative treatment for tonsils and adenoids, 7s. 6d.; diseases of women, 3s. per day; diseases of children, 1s. 6d. per day.

There would be additional charges for dentures (cost price); surgical instruments (cost price); spectacles, 2s. 6d.; and repairs to spectacles, 1s.

The Council to consider the remission of hospital fees and additional charges, but not the annual registration fee in cases whose circumstances so warrant.

### AN ESTIMATED REVENUE OF £2,362.

Dealing with the finance of this proposal, the Medical Officer assumes that on account of the charges the number of persons attending the clinics will diminish by 25 per cent. Hence he computes that the following would be the additional income accruing from these proposals for the year 1921-22, assuming the fees become operative on October 1: Registration, £1,004; confinements, £522; hospital treatment of women, £68; hospital treatment of children, £458; dentures, £300; surgical instruments, £50; spectacles, £110. Total, £2,512, of which £150 would be required for expenses, etc., in regard to registration and clerical work involved.

Regarding power to fix such an arrangement, the Medical Officer says it would appear to be at the discretion of the Council to decide whether a charge is made or not for maternity and child-welfare cases. As far as school cases are concerned, medical inspection and treatment are both compulsory on the Council. Whether it is possible for any charge to be made for routine medical inspection at the schools is questionable, as it is the duty of the local education authority to provide for this; but with regard to medical treatment at the clinics a charge would appear to be in order.

Dr. Buchan explains that the charges have been arranged as far as possible so that their payment shall not operate to keep patients away from the clinics until they are ill. The principle governing the institution of health clinics is to see people when they are well in order that illness as far as practicable may be prevented in the mother or the child. There may be difficulties in connection with school cases referred by teachers. Hitherto all school cases referred by head teachers have been seen and advice given or remedial measures adopted. Where a system of payment is in operation it will not be practicable to see patients referred direct by teachers. Such cases would have to be first visited at the home, and arrangements made as to payment of the registration fee. The acceptance of an annual registration fee would imply a contract of service, and the services available would have to be specifically stated with some form of reservation, so that the Council might be in a position to veto any demand which might be made.

